# The Lived Experiences of Saudi Nursing Students in Digital Clinical Experience: A Phenomenological Study

**DOI:** 10.7759/cureus.53830

**Published:** 2024-02-08

**Authors:** Bander Albagawi, Yasir Alsalamah, Maryam Alharbi, Rakan Alrawili, Lisa A Babkair, Rabia Allari, Sara Alkharji, Reham Abed, Mirna Fawaz

**Affiliations:** 1 Nursing, University of Hail, Hail, SAU; 2 Nursing, Mental Health Hospital, Qassim Health Cluster, Qassim, SAU; 3 Nursing, Faculty of Health Science, University of Pretoria, Pretoria, ZAF; 4 Pediatrics, Hematology-Oncology, King Abdulaziz Medical City, King Abdullah Specialized Children's Hospital, Riyadh, SAU; 5 Health Affairs, Academic and Training, Ministry of Health, Aljouf, SAU; 6 Critical Care Nursing, Faculty of Nursing, King Abdulaziz University, Jeddah, SAU; 7 Acute & Chronic Care Nursing, Faculty of Nursing, Al-Ahliyya Amman University, Amman, JOR; 8 Mental Health, Nursing Affairs, King Saud University Medical City, Riyadh, SAU; 9 Genetics, King Saud Medical City, Riyadh, SAU; 10 Nursing, Beirut Arab University, Beirut, LBN

**Keywords:** nursing students, shadow health, digital clinical experience, nursing education, simulation

## Abstract

Background

Novel digital methods of simulation are gaining popularity in nursing education in light of the limited access to clinical placement and expensive high-fidelity simulation technologies.

Aim

The aim of this study is to explore the lived experiences of Saudi nursing students in digital clinical experiences (DCEs).

Methods

A qualitative phenomenological research design, grounded in Husserlian phenomenology, was employed. Purposive sampling was utilized to select 21 participants actively involved in DCEs. In-depth interviews were conducted to collect rich, narrative data.

Results

The thematic analysis has yielded four themes, namely, “comfort and safety”, “critical thinking and problem solving”, “appraisal of knowledge”, and “transition to practice.”

Conclusions

The findings contribute to ongoing discussions about leveraging technology in nursing education, emphasizing the need for educators and policymakers to integrate digital tools that enhance the learning experiences of nursing students.

## Introduction

A multimodal strategy is necessary to assist student nurses acquire information in their nursing studies. In order to meet learning objectives intended to train future nurses, nursing concepts and expertise are utilized in clinical learning scenarios. Problem solving, competency, management, interpersonal skills, clinical nursing, and compassion are all included in hands-on, context-specific, and interactive experiences guided by learning objectives [1]. In order to deliver safe, high-quality care, nursing students must acquire the understanding required to meet the specified learning goals. The ultimate objective is to seamlessly move from an undergraduate student to a certified professional [2]. In the past, the goal of nursing programs was to attain student learning and certification goals by combining two learning settings: practical education in healthcare facilities and conventional theoretical instruction in class. These two instructional settings have been adjusted lately in response to modifications in the training of nurses. For instance, a growing interest in interactive education, demographic disparities between professors and undergraduate populations, distance education, and computerized instructional resources have all had an impact on conventional pedagogic teaching [3]. The increasing quantity of nursing schools and learners, the decline in clinical opportunities, the restrictions placed on what students may perform, and the lengthy certification processes in numerous clinical settings have all had a bearing on field placements.

For a period of time, digital clinical experience (DCE) has replaced conventional encounters within healthcare and facility-based settings as a result of the COVID-19 outbreak [4]. Through the completion of several patient care tasks, trainees are given consistent patient encounters through the Shadow Health® Digital Clinical Experience^TM^. This entails gathering both objective and subjective patient information, using therapeutic interaction techniques, and developing treatment strategies. Nursing learners can interview, evaluate, record, and report on their experiences with the Shadow Health® digital standardized patient using the online virtual patient simulation known as the Shadow Health® Digital Clinical Experience^TM^. According to Wang et al. [5], DCE is structured into a number of consecutive components that represent the many elements of a health evaluation, such as obtaining a medical history and performing a head-to-toe assessment [5].

It has been established that offering undergraduate nurses consistent clinical scenarios using DCE is both engaging and affordable. By giving every learner the same simulation setting, virtual patients establish an impartial educational setting. Compared to standardized patient performers who may introduce flaws or get tired while repeating an identical scenario with several students, DCE gives learners a more consistent option to evaluate their abilities [6]. Virtual patients also have the benefit of being more accessible for more independent time-flexible simulation encounters, thereby rendering them suitable for distant learning programs. Although DCEs are somewhat recent in the realm of medical simulations, they are attractive in the discipline of nursing academia, in which there is a dearth of instructors and funds are usually restricted, due to their inexpensive nature when compared to models employing high-fidelity simulators or patient performers. Furthermore, relative to no simulations, digital clinical experiments have been demonstrated to provide substantial advantages in terms of learning outcomes. Numerous clinical domains have benefited from the utilization of virtual patients in healthcare occupational training [7]. The improvement or evaluation of critical thinking abilities is one of the learning goals for virtual patient simulators. Research on the experiences of learners with this simulation mode does, however, suffer from a notable scarcity. Thus, the purpose of this study is to explore the lived experiences of Saudi nursing students taking part in Shadow Health® Digital Clinical Experience^TM^, namely, in the health assessment, medical-surgical, pediatrics, and community health modules. The specific objectives of the study include identifying the perceptions of Saudi nursing students regarding the DCE and exploring the impact of the DCE on the overall learning outcomes and transition to practice among Saudi nursing students.

## Materials and methods

Research design

Given the exploratory nature of the research questions and the emphasis on capturing rich, contextual data, a qualitative methodology is deemed most suitable. Through in-depth interviews and open-ended questioning, the study intends to gather detailed narratives that provide a comprehensive understanding of the phenomenon under investigation. This study adopts a phenomenological research design, specifically drawing from the philosophical framework developed by Husserl [8]. The phenomenological approach, as utilized in this research, seeks to uncover and interpret the subjective experiences of Saudi nursing students within the context of digital clinical experiences. This approach enables a deep exploration of the meaning and essence of these experiences as lived by the participants. As a result, constructivism serves as the research's epistemological stance, acknowledging the existence of various realities and the examination of participants' experiences via their social interactions with the environment [9]. This article was developed in accordance with the consolidated criteria for reporting qualitative research (COREQ) principles.

Setting and sampling

The study was carried out at one university in Saudi Arabia, which offers a four-year bachelor's nursing program and is affiliated with multiple university hospitals. The nursing department at the approached university is actively engaged in simulation activities, where it offers high-fidelity simulation experiences and standardized patient programs to nursing students starting the first semester of the first year, in the practical aspect of the curriculum. However, after the COVID-19 pandemic, the nursing department started looking into digitalized experiences that could even be used in distance learning mode.

A purposive sample with maximum variation sampling was sought in order to recruit an internally heterogeneous and externally homogenous group of students who had lived experience with DCE. This was achieved by recruiting students with a range of ages, genders, and levels of the program (students from first, second, third, and fourth years registered in different courses). Out of 28 students who were eligible to participate in the study, 21 students responded, which yielded a response rate of 75%. This sample size was used considering we had students from various levels of the program.

Inclusion criteria

To be eligible to participate, students should be actively registered in either of the academic levels of the program (first- to fourth-year undergraduates), have no prior professional experience in nursing, and did not enroll previously in any technical nursing program or technical nursing acceleration program. Otherwise, they would be excluded from the study.

Digital Clinical Experience^TM^

The nursing program that the participants were enrolled in is designed in a way, where each course has a theoretical, practical, and clinical aspect. The theoretical part provided the students with theoretical knowledge and the practical part engaged students in simulation and laboratory sessions; as for the clinical part, it engaged the students with actual clinical placement in the hospitals/clinics to practice what they have learned in the real setting. These students participating in this study received the practical component of one theoretical course through DCE before being transferred to clinical placement pertaining to that course. First-year students received the health assessment package, second-year nursing students received the medical-surgical package, third-year nursing students received the pediatric package, and fourth-year nursing students received the community health package. The use of various modules of shadow health enabled aligning the DCE with the current level of students and the intended learning outcomes needed to be covered within the registered courses. The modules focused on clinical skills, as well as higher-order thinking skills such as critical thinking and clinical reasoning. The students were not obliged to engage in Shadow Health® Digital Clinical Experience^TM^, as this was a voluntary activity that was suggested to the students in different classes. Thus, the students’ official grades were not affected by their participation in this experience. However, the DCE was used as a formative activity. Considering that this was the students’ first experience with Shadow Health® Digital Clinical Experiences^TM^, the students were familiarized by their nursing instructors with the software through orientation and debriefing sessions. During the scenarios, the nursing instructors made sure to stay around to troubleshoot any emergent problems. Considering that the official language of instruction in the nursing program is English, the students had no problems with interacting with patient scenarios in the DCE. The administration of the nursing department made sure to buy the product as part of the enhancement of the teaching and learning modalities being tested and adopted at the department. Thus, students had no expenses to cover.

Recruitment and data collection

A skilled research assistant contacted the students in between lectures to invite them to take part in the study, taking into account that the students had previous interactions with some of the investigators when they were teaching nursing courses at the university. In this manner, any potential imbalance in power would not have resulted in a factor of compulsion affecting the students' decision to give consent. The research assistant involved in this study works as an administrator at the department and already has access to the list of registered students. Thus, student registration records were not shared with any external party, thus maintaining the confidentiality policy of the university. The research assistant gave the students a fact sheet and described the purpose of the inquiry. The fact sheet precisely specified to the students that participation was voluntary and anonymous. The researchers made sure that no personal identifying data were collected or published and that the interviews were to be conducted in a private space. Individuals who expressed interest in taking part completed a written informed consent form, sent it in via email, and arranged for a private, convenient one-on-one interview. The period of data collection was extended to September and November 2023.

Interviews

One investigator, who was not involved in teaching the students participating in this study, performed a semi-structured interview. The investigator has a Ph.D. and is familiar with qualitative research and interviewing techniques. For the same considerations as previously mentioned, the university-affiliated researcher did not perform the interviews. To ensure the respondent's convenience, the interviews were performed in each participant's naturalistic environment and on their own time. During the conversations, the interviewer avoided any assumptions by practicing reflexivity. The investigator also made sure not to wear a lab coat or a suit and used spatial proxemics in a way that enabled a comfortable conversation and prevented intimidating the students by not displaying any characteristics of power. Each interview took 45 minutes to one hour, with the investigator spending time alone with the respondents to ensure they felt comfortable sharing their experiences and opinions. The investigator gave a brief introduction before restating the main goal of the conversation. He informed the respondents that all conversations would remain private and anonymous and reiterated consent for the interviews to be audio recorded. Field notes were obtained throughout the interviews in order to consider any nonverbal clues and contextual details that could be relevant to the study. The interview guide by Krueger et al. [10] was utilized, which included introduction, transitional, and closing questions. When necessary, probing questions were employed, and by the time the interview was over, the students were asked closing prompt questions (Table 1).

**Table 1:** Interview Questions

Category	Questions
Introduction Question	Can you introduce yourself to me? What are your thoughts on “simulation” and “digital clinical experiences”?
Transition Question	What were your impressions of the first time you engaged in DCE?
Key Questions	How do you feel about your experience with DCE?
	How would you compare it to clinical practice?
	How do you think it affected your clinical practice?
	What kind of things did you learn?
Final Prompt	Do you have anything further to say?
Probing Questions	Could you give us a better description?
	Could you provide us with a better description?

Data analysis

The researcher who performed the interviews carefully transcribed the conversations after listening to the audio tapes several times. Pseudonyms were assigned to the students to maintain confidentiality and anonymity of the students. The study team was then given the transcripts to review. The seven stages of inductive thematic analysis - reading and revisiting the transcripts, coding important phrases, establishing meanings, creating groupings and themes, developing themes, offering thick descriptions, and confirming the results with the participants - were applied [11]. To maintain the conclusions firmly based on the voice of the students and to counter the team members' presumptions, analyst triangulation was used by having three members of the team analyze the data. The three members of the analysis team thoroughly examined the data, independently coded, and created preliminary themes. The three team members then got together, talked over the results, and worked out any disagreements. To make sure the three members of the analysis team fully understood the students' opinions, the verbatim transcription was reread many times. Next, an appropriate term was assigned to each quote, emphasizing the true significance of the offered data. Following the clustering, rearranging, and consolidation of these terms into qualitative themes, the investigators closely scrutinized the themes to make sure they appropriately reflected the experience that was the subject of the study. Field notes were used during the interviews to capture any non-verbal cues and were considered during the analysis of the transcripts. However, no pertinent data were noted.

Trustworthiness and credibility

To guarantee the rigorousness of the findings, the five-step trustworthiness criteria - credibility, dependability, confirmability, transferability, and authenticity - proposed by Guba et al. [12] were employed. Using member-checking will improve the trustworthiness of the findings. Giving participants the pre-established categories and descriptions enabled us to determine whether or not they still fairly reflect the respondents' points of view. Throughout the data processing phase, utilizing several coders helped increase the conclusions' legitimacy [13]. This will test the assumptions made by the investigators and guarantee that the participants' statements match their results. Dependability and confirmability were enhanced by creating an audit trail that includes raw data and field memos recording observations, perceptions, remarks, process notes, and the underlying presumptions of analytical judgments. Providing the features of the intended sample and providing thorough explanations of the respondent's narratives can improve transferability. These comprehensive illustrations and the inclusion of quotes from respondents in the results helped fairly and correctly portray the variety of individual opinions. Authenticity emphasizes how important it is to give participants a voice, enhance individual beliefs, and inspire initiative via the sharing of study findings.

Ethical considerations

The Institutional Review Board authorization was given by the university (Umm Al Kura University) where the data were collected (IRB number: ECO-R-201). Following their assurance that their participation in the research was completely optional and that withdrawing from it would not have any negative consequences, all respondents willingly gave written informed consent. Additionally, it was repeatedly stressed to the students that their participation would remain confidential and that no private data would be included in any published content. To maintain anonymity, each responder was given a pseudonym, and the transcripts were anonymized and encrypted. The recordings and transcriptions were stored in a password-protected folder that was only accessible to the study team.

## Results

Characteristics of the respondents 

The sample of the study comprised 21 students in total, where 11 were males (52.4%) and 10 females (47.6%). The average age in the sample was 22.7 years. The participating students were given pseudonyms to maintain anonymity. The pseudonyms are denoted by the letter S (signifying student) followed by a number.

Qualitative findings

Comfort and Safety

It was repeatedly noted by participants that they felt comfortable and flexible when adjusting to the virtual clinical setting. This flexibility showed itself in a number of ways, including psychological adjustment, technological navigation, and realistic yet simulated patient care scenarios. First of all, a common observation among participants was their quick learning curve for using the digital tools and interfaces unique to the virtual clinical setting. "I was astonished at how quickly I became accustomed to the software," said one of the pupils. "After a few times, it turned into routine. It's a distinct level of skill that gives our nursing education something extra..." (S18). Another participant remarked "...it felt like entering into a well-constructed process where each action felt logical...Moving into the digital patient scenario was very simple. It became easy to navigate through the situations, transforming what once appeared to be a complicated learning process into a well-planned educational experience..." (S3). The students participated in reflective activities, emphasizing their deep comfort level in the autonomous learning environment in specific. This special feature of their education gave them opportunities for independent study and gave them a sense of freedom and control that greatly improved their overall contentment and academic results. One student said, for instance, "…the time flexibility provided by digital clinical encounters is one of their biggest benefits. It has changed everything for me to be able to learn at my own speed. I can study topics, go over instances again, and fully comprehend the information without feeling pressed for time. It's similar to receiving individualized instruction that meets my requirements and deepens my comprehension of patient care…" (S10). Another narrative stated "Learning at my own pace has been immensely freeing...there's always a hurry in conventional settings, but with digital clinical encounters, I can choose when to really engage in the learning process. It's important to identify the best times for me to take in the intricacies of nursing practice..." (S4). Another statement said, "For me, digital clinical experiences have completely changed how convenient nursing education is...I can fully engage myself in comprehending patient care without getting overwhelmed ...It's important to prioritize quality over quantity, and convenience further deepens my learning process..." (S21). Students stressed the need to provide a secure learning environment in the digital clinical setting that goes beyond the conventional classroom and medical environment. In addition to being physically secure, this safety also includes psychological comfort, enabling learners to explore, make errors, and learn without worrying about criticism or repercussions. One student remarked, for instance, "The virtual environment is a safe place for learning; it's more than just scenarios...This is a safe haven where I may learn and experiment without fear of real-life consequences for my failures." Another student declared "Practicing on virtual patients eliminates the anxiety, ...realizing that I can make mistakes, grow from them, and improve my techniques without worrying about hurting a real patient is a comfort…mental security is priceless..." (S15).

Critical Thinking and Problem Solving

Participants in the present investigation explained how their dynamic participation was stimulated by digital clinical encounters. Because these simulations are interactive, it is necessary to use conceptual understanding, in addition to critical thinking skills and situational adaptation. One student said, for instance, "We actively participate with realistic medical scenarios in this unique environment. It's as though you've entered the actual nursing environment, where choices are important and patient care is complex. Every contact in the simulated scenarios tests our ability to think critically, assess the circumstances, and move quickly, prepping us for the unpredictability of patient interactions..." (S7). "The settings change, and we have to change, making choices that have instant effects," remarked a different student." "Our schooling is greatly aided by this dynamic activity, which helps us improve our critical thinking abilities in a manner that conventional approaches are unable to fully convey" (S13). The respondents stated that students were able to smoothly transition from theoretical principles to practical applications owing to the digital clinical setting. Students had to engage in more sophisticated critical thinking, which required them to make connections, form conclusions, and use what they learned in a clinical context. For example, one student exclaimed, "They enable us to effortlessly translate theoretical concepts into everyday life... acquiring knowledge is not enough; one must also comprehend how those details are used to patient care... virtual situations provide a link between the changing realities of nursing practice and the concepts we learn in textbooks" (S20). A participant also mentioned "...the virtual situations are a genuine, breathing representation of the content we gain in lectures rather than anything apart from it....it's the Aha moments when we understand how theory influences our decision-making in the complicated patient settings and the real-world applications of what we've learned" (S9). Additionally, the students said that the digital clinical environment gave them an opportunity to work through issues and find solutions even in the face of ambiguity. The scenarios frequently provide unforeseen difficulties, forcing students to improvise, weigh their alternatives, and come up with workable answers. One of the stories went something like this, "...our problem-solving abilities really come forward when we are faced with unknowns, as a result of our digital clinical encounters...finding viable options in the changing patient care circumstances, managing through the unknown, and adjusting to unforeseen problems are more important..." (S2). As said by a different student, "It was evident how to solve issues in the digital clinical setting. The cases test our ability to think strategically, assess problems quickly, and develop strategies that deal with the variables and complications... The simulation replicates the practicalities of our upcoming work" (S11).

Appraisal of Knowledge

Through an introspective method, the participating students discovered that they were evaluating their prior knowledge. Students discovered their strengths, places for growth, and ways to enhance their patient care methods through this contemplation, which acts as a catalyst for both personal and professional development. One student declared, for example, "…I get to add something new to my learning every time I interact with a virtual patient. Evaluating past knowledge is a continuous, active activity rather than only a look back. It all comes down to transforming such encounters into turning points in my drive to become a more skilled and sympathetic nurse…" (S1). Furthermore, the students stated that they improved their clinical skills, decision-making ability, and general competency in the virtual context by evaluating prior information." "The digital clinical encounters are like a polishing laboratory for our practical skills...every circumstance serves as a blank canvas on which we can exercise our abilities...it involves deliberately fine-tuning our method of patient care, making sure that every choice is better informed and each action is more accurate than the last, rather than merely repeating acts..." was an excerpt of one of the shared experiences (S6). A further narrative was "We aim for mastering; we are not satisfied with just knowing...in order to improve our abilities, make more complex clinical judgments, and make sure that our skill set is a changing, constantly developing thing, we consciously evaluate our prior knowledge..." (S17). Additionally, students said that they actively participated in self-directed learning in an effort to fill up the information gaps that were discovered throughout the assessment process. One of the prevalent views, for instance, was "We now have a habit of pursuing knowledge exceeding what is given to us in the cases as a result of our interactions with virtual patients... it’s a form of continuous dedication to lifelong learning... it's important to develop a mindset that values ongoing inquiry and views every situation as a starting point on our never-ending educational path, rather than just passing tests…” (S14).

Transition to Practice

The last theme sheds light on the transforming process that students go through as they learn to make the connection between the complexity of real-world clinical practice and instructional simulations. During the interviews, the students emphasized how important it is to employ the abilities they have developed in virtual clinical experiences in real-world clinical situations. One student put it this way: "I've trained and improved my abilities... it's like a boot camp...it's now my responsibility to put those practiced abilities to use in actual clinical situations when I enter them. There is a change from the virtual to the actual, where each action has actual repercussions and each ability serves as an important instrument for patient care..." (S4). Students also emphasized how critical it is to use information and abilities flexibly in unforeseen circumstances in order to prepare for the dynamic and sometimes unpredictable character of medical care." "I gained experience adapting to the limitations of a scenario in the digital clinical setting...It's about moving from the familiar to the unknown, where my capacity for adaptation serves as a guide to help me navigate the challenges of providing real-world patient care...It's similar to going from a theatrical script that is organized to the spontaneous improv needed to deal with unforeseen obstacles in the treatment of patients" (S15).

## Discussion

An array of clinical judgment abilities, including structured evaluation of patients, critical thinking, and relational abilities to extract the salient features of patient manifestations, motor abilities to enable practical examinations, and emotional abilities to support these procedures, must be developed and practiced by nursing students [14]. Simulation is a crucial part of nursing schooling because it provides a prompt, secure, and directed way to accomplish learning goals across various course levels [15]. A number of teaching benefits that actual patient interactions cannot provide are provided by simulation, such as the ability to repeat situations, concentrate on certain illnesses and situations, and provide a safe setting in which students may make errors without endangering patients [16]. Accordingly, as the sector works to produce professionals who are ready for practice, simulation has developed into a significant part of the curriculum for bachelor-level nurses [17].

This study aimed to explore the perceptions of student nurses in a novel model of simulation, namely, virtual patient simulation by the Shadow Health® Digital Clinical Experience^TM^. The results of this study found that nursing students perceived that DCE boosted the students' critical thinking, problem solving, and confidence skills. It helped them navigate critical patient care situations that would have made them highly anxious in the real clinical setting. It is challenging to compare results since there are few published research examining Shadow Health® Digital Clinical Experience^TM^ in nursing research. For a number of factors, it has been discovered that DCEs are either better or similar to other high-fidelity conventional simulation approaches. Our findings resonate with those of Duff et al. [18] who conducted a comprehensive evaluation of 12 research papers put out between 2008 and 2015 and discovered that, with regard to improved student learning, contentment, and involvement, DCEs were on par with or better than conventional simulation techniques to reinforce clinical reasoning and evaluation abilities. In addition to their other advantages, Duff et al. [18] found that these simulation experiences were more difficult and genuine than those created with physical simulators or standardized patients because they could be created virtually and included physiological data that could not be replicated by standardized patients [19-22].

A comprehensive investigation on the efficacy of DCEs in education for health professionals was carried out by Kononowicz et al. [23]. The scenarios were contrasted with conventional instruction, mixed with conventional instruction, and other forms of computerized education. Results showed that DCEs can certainly significantly increase learning results and more efficiently develop a variety of abilities as opposed to conventional instruction. DCEs have a good influence on a variety of nursing educational outcomes for students, according to Foronda et al. [24], who conducted a comprehensive review covering more than 20 years of published research. The majority of studies demonstrated that DCE was a useful pedagogy for enhancing learning, skills, reasoning skills, self-assurance, and learning pleasure. In addition, in several investigations, intellectual and psychomotor acquisition effects from DCEs were comparable to those from regular high-fidelity simulation experiences. Similarly, other research [19,20,25,26] has discovered that learners become more involved with DCEs and appreciate having a secure atmosphere to hone their critical thinking abilities before interacting with actual patients in a hospital. Students can get immediate input that is more closely related to the skills they are practicing by employing DCEs that run asynchronously [19]. By questioning students' preconceived notions and understanding in light of a more profound and significant patient engagement, DCEs may offer them life-changing educational opportunities [27]. The impacts of taking part in DCEs were assessed by Brown et al. [28] in a quantitative cross-sectional research with undergraduate student nurses. These students gave the DCE's usefulness an overall above-average rating. These results validated the use of DCEs as a useful method for providing hands-on experiences. However, students studying pharmacy did not show a statistically noteworthy rise in self-assurance following DCEs, according to Taglieri et al. [29]. However, they did observe a decrease in self-assurance following the initial experience and a boost following the subsequent one. Taglieri et al. [29] believe that interaction with and access to DCEs may account for the recovery of self-assurance to the starting point and that the first fall may be related to an exaggeration in the rating of self-assurance.

Limitations

A phenomenological descriptive approach was used at one university in Saudi Arabia in this research, which prevents findings from being transferred to other settings. For the first time, though, the real-life encounters of nursing students who participated in DCE were examined. This might be a significant addition to the global discussion over DCE's efficacy in enhancing educational results for bachelor-level nursing students.

## Conclusions

In conclusion, this phenomenological study has revealed a multifaceted perspective, highlighting the positive impact of these virtual encounters on perceived students' confidence, comfort levels, and the acquisition of critical thinking and problem-solving skills. The virtual environment appeared to foster a safe space for learning, allowing students to practice and refine their skills without the constraints and pressures of a traditional clinical setting. The virtual platform provided a flexible and accessible learning environment, accommodating the diverse needs of the students and contributing to an overall positive educational experience. This comfortable yet stimulating and engaging aspect in the digital realm has implications for the future of nursing education, suggesting that integrating technology into clinical training can enhance the overall learning experience for students. Further studies are still needed to build a body of evidence regarding the investment of nursing educational programs in this simulation modality in combination with other high-fidelity simulation programs. This warrants comparative studies of learning outcomes between programs applying Shadow Health® Digital Clinical Experience^TM^ and those that do not. Furthermore, in future qualitative inquiries, it would be interesting to study the challenges that might arise and the further application of this learning modality.
